# The compensatory reserve index predicts recurrent shock in patients with severe dengue

**DOI:** 10.1186/s12916-022-02311-6

**Published:** 2022-04-07

**Authors:** Huynh Trung Trieu, Lam Phung Khanh, Damien Keng Yen Ming, Chanh Ho Quang, Tu Qui Phan, Vinh Chau Nguyen Van, Ertan Deniz, Jane Mulligan, Bridget Ann Wills, Steven Moulton, Sophie Yacoub

**Affiliations:** 1 Hospital for Tropical Diseases, Ho Chi Minh City, Vietnam; 2 Oxford University Clinical Research Unit, 764 Vo Van Kiet, District 5, Ho Chi Minh City, Vietnam; 3 University of Medicine and Pharmacy at Ho Chi Minh City, Ho Chi Minh City, Vietnam; 4 Centre for Antimicrobial Optimisation, Imperial College London, London, UK; 5 Sierra Nevada Corporation, Sparks, NV USA; 6 Context Data Analytics Ltd, Longmont, CO USA; 7 Centre for Tropical Medicine and Global Health, Nuffield Department of Clinical Medicine, Oxford University, Oxford, UK; 8 Department of Surgery, University of Colorado School of Medicine, CO Aurora, USA

**Keywords:** Dengue, Shock, Re-shock, Compensatory reserve index (CRI), Non-invasive monitoring, Pulse waveform, Machine learning

## Abstract

**Background:**

Dengue shock syndrome (DSS) is one of the major clinical phenotypes of severe dengue. It is defined by significant plasma leak, leading to intravascular volume depletion and eventually cardiovascular collapse. The compensatory reserve Index (CRI) is a new physiological parameter, derived from feature analysis of the pulse arterial waveform that tracks real-time changes in central volume. We investigated the utility of CRI to predict recurrent shock in severe dengue patients admitted to the ICU.

**Methods:**

We performed a prospective observational study in the pediatric and adult intensive care units at the Hospital for Tropical Diseases, Ho Chi Minh City, Vietnam. Patients were monitored with hourly clinical parameters and vital signs, in addition to continuous recording of the arterial waveform using pulse oximetry. The waveform data was wirelessly transmitted to a laptop where it was synchronized with the patient’s clinical data.

**Results:**

One hundred three patients with suspected severe dengue were recruited to this study. Sixty-three patients had the minimum required dataset for analysis. Median age was 11 years (IQR 8–14 years). CRI had a negative correlation with heart rate and moderate negative association with blood pressure. CRI was found to predict recurrent shock within 12 h of being measured (OR 2.24, 95% CI 1.54–3.26), *P* < 0.001). The median duration from CRI measurement to the first recurrent shock was 5.4 h (IQR 2.9–6.8). A CRI cutoff of 0.4 provided the best combination of sensitivity and specificity for predicting recurrent shock (0.66 [95% CI 0.47–0.85] and 0.86 [95% CI 0.80–0.92] respectively).

**Conclusion:**

CRI is a useful non-invasive method for monitoring intravascular volume status in patients with severe dengue.

**Supplementary Information:**

The online version contains supplementary material available at 10.1186/s12916-022-02311-6.

## Background

Dengue is the most important mosquito-borne viral illness in humans. It continues to spread to new areas around the world and affects more than 100 countries, due to globalization and climate change [1]. Dengue is responsible for approximately 390 million infections and about 20,000 deaths annually [2]. Although the majority of dengue patients recover, about 5% will develop potentially lethal complications during the critical phase, around day 4–6 of illness. These complications include bleeding due to coagulopathy, multi-organ impairment, and/or severe plasma leakage leading to dengue shock syndrome (DSS) [3]. There is no specific treatment for dengue, other than optimal supportive care [4].

Carefully adjusted fluid resuscitation is required for patients who develop DSS, particularly during the initial 24–48 h after the onset of shock. About one third of these patients will experience one or more episodes of shock due to severe ongoing plasma leakage, and many will require significant amounts of fluid, resulting in respiratory distress and poor outcomes [5]. The prediction of this re-shock or recurrent shock in dengue is important in order for clinicians to provide closer monitoring and enact appropriate and timely interventions. Delayed recognition of re-shock increases the risk of multi-organ failure, poorer health outcomes, and death. It remains extremely challenging for clinicians to accurately determine intravascular volume status and identify the subset of patients who will develop recurrent episodes of shock. Prognostic models have been developed to help identify dengue patients who are likely to have poor outcomes and include parameters such as age, illness day, heart rate, and temperature [6]. These parameters are not, however, dynamic nor do they account for individual variability or changes in hemodynamic status over time during fluid resuscitation. Heart rate, blood pressure, and hematocrit are the current gold standards for monitoring patients with DSS, but are relatively insensitive and change late, oftentimes just minutes prior to an episode of re-shock [4]. A narrow pulse pressure (≤ 20 mmHg) accompanied by reduced peripheral perfusion is an indication of compensated shock and part of the WHO definition of DSS [7]. There is a large, unmet need for dynamic monitoring tools, especially non-invasive methods, to predict shock or re-shock—which is of particular importance in severe dengue due to the associated coagulopathy.

The compensatory reserve Index (CRI) is a novel vital sign parameter that uses machine learning derived features of the pulse arterial waveform, or photoplethysmography (PPG), to reflect real-time changes in intravascular volume. The pulse arterial waveform is processed by an algorithm to calculate CRI with a value between 1 and 0, where 1 indicates normovolemia and 0 represents the point of decompensated shock (SBP < 80 mmHg) [8]. The CRI score was developed using a simulated model of hypovolaemia and, as a result, is able detect early physiologic changes that occur during the compensatory phase of central volume loss, preceding changes in conventional hemodynamic parameters [9–11]. CRI has also been shown to be individual specific; the algorithm has learned to identify individual variability to compensate for fluid loss [12, 13]. In dengue patients, a preliminary case series assessing CRI in three children with DSS demonstrated potential utility at tracking changes in intravascular volume [14]. In the current study, we investigated dynamic changes in CRI in intensive care unit (ICU) patients with DSS to determine if CRI could predict episodes of re-shock. We hypothesized that the CRI algorithm would identify patients at risk for developing re-shock, prior to changes in traditional vital signs.

## Methods

Patients were enrolled in a prospective observational study and we selected a subgroup of patients with DSS among 103 hospitalized patients in the pediatric and adult intensive care units at the Hospital for Tropical Diseases (HTD) in Ho Chi Minh City, Vietnam. The full methods for this study have been published elsewhere [11]. Briefly, the inclusion criteria for the study are individuals above 3 years admitted to either pediatric or adult intensive care unit with a clinical diagnosis of severe dengue or with multiple warning signs at high risk of developing shock. Clinical and laboratory data were collected daily for a maximum of 5 days from enrolment and at follow-up 2 weeks later. Laboratory data included daily full blood count, along with liver enzymes and renal function at three time points. Detailed hemodynamic assessments were performed with hourly vital signs, urine output, fluid volume, and type of fluid given, in addition to any other interventions such as inotrope use. All patients were managed using hospital fluid management guidelines. Dengue severity was classified according to the WHO 2009 guideline. Ethical approvals were obtained from the Oxford Tropical Research Ethics Committee and the Ethics Review Committee at HTD. Written informed consent was obtained from all participants or a parent/guardian.

### Dengue diagnosis

A commercial IgM and IgG assay (Capture ELISA, Panbio, Australia) was used on acute and convalescent plasma. RT-PCR was done to identify serotype and viremia levels. Confirmation of dengue diagnosis was defined as either being positive by IgM or RT-PCR.

### Compensatory reserve index

CRI was measured by fingertip pulse oximetry and continuously collected from enrolment until discharge, or for a maximum of 5 days. The PPG data was wirelessly transmitted via Bluetooth to a bedside laptop computer and synchronized with the patient’s clinical data. Pulse waveform data was converted to CRI values by a research scientist at Flashback Technologies, Inc., in the USA after the data collection period was completed, who was blinded to all clinical data. All clinical data (vital signs, fluid volumes, organ support and urine output) were entered into an electronic medical record (MEDICS, Sierra Nevada Corp), specifically designed for the study. Summary of outcomes and laboratory tests were recorded using paper case report forms (CRF) and integrated with digital data in each patient’s electronic medical record.

### Statistics

Patients who developed DSS around the time of enrolment in the ICU were included in the analysis. We excluded those who were enrolled but did not subsequently develop DSS developed DSS late after enrolment and those without sufficient clinical or CRI information. DSS was diagnosed if the pulse pressure was ≤ 20 mmHg, accompanied by signs of poor peripheral perfusion (cold extremity and/or capillary refill time (CRT) ≥ 2 second). Re-shock was defined as a separate episode of DSS which occurs at least 6 h after the clinical resolution (defined as pulse pressure > 25 mmHg without signs of impaired perfusion) of an initial episode of dengue shock. Both DSS and re-shock are clinical endpoints which are provided by the attending physician caring for the patient. All episodes of re-shock occurred within 48 h from enrolment; only data within this period of time were used for data analysis. A formal sample size calculation was not possible given the CRI represented a novel measurement modality with limited experience in its implementation, and patients were recruited over 2 dengue seasons as a pragmatic cohort.

The CRI monitor provides beat to beat CRI values; however, to avoid erratic variations we used an averaged CRI value over 5 min. Individual trajectories of CRI were plotted over the first 48 h, with systolic and diastolic blood pressure, and the amount of fluid received overtime.

Descriptive summaries were performed using median (interquartile range) for continuous variables and frequency (percentage) for categorical variables. Graphical descriptions of CRI and hemodynamic parameters are based on a time-plot of the individual trajectory of the parameter of interest. Correlations between CRI and hemodynamic parameters were assessed using partial Pearson’s correlation coefficient, controlled for potential confounding variables including age, gender, and body weight. CRI and hemodynamic data were grouped either every 30 min (for CRI and heart rate) or 1 h (for CRI and blood pressure) from onset of first shock. The significance of partial correlations was based on their Fisher transformation and the bootstrap standard errors, using 1000 resamples to account for repeated data. All CRI data obtained after recovery from the first episode of shock and within the first 48 h from enrolment were used to investigate CRI’s ability to predict re-shock. For each CRI measurement, we derived the occurrence of first re-shock within 12 h from its measurement as primary outcome and the occurrence of pulse pressure ≤ 20 mmHg as secondary outcome. These analyses were based on logistic regression models, with and without adjustments for time of CRI measurement, age, gender, and body weight. The ability of the CRI to predict outcomes was quantified using area under the ROC curve analyses, as well as sensitivity and specificity at different CRI cutoffs (0.2, 0.4, 0.6). To account for repeated measurements per individual, all confidence intervals for OR, AUC, sensitivity, and specificity were calculated using the cluster bootstrap standard errors based on 1000 resamples of the original dataset. In addition, CRI and HR measurements were grouped and displayed at different 1-h windows before the first re-shock (for patients with re-shock) and after enrolment (for patients without re-shock). All analyses were performed with R version 4.0.3 [15], in companion with ROCR [16], and ggplot2 packages [17].

## Results

A total of 103 patients were enrolled. After removing non-DSS cases (patients admitted to an ICU only for monitoring) and DSS cases without CRI data, there were 63 total cases (50 children and 13 adults) for the final analysis (Additional file 1: Fig. S1). Clinical characteristics are described in Table 1. The median age was 11 years (IQR 8–14), and there were similar proportions of male and female patients. Five (8%) patients exhibited severe shock at the time of admission (four had pulse pressures ≤ 10 mmHg, and one had no measurable blood pressure). The re-shock rate was 24% and 3 patients required inotropic support. Respiratory distress occurred in eight patients (13%); however, no patient required ventilatory support. There were no deaths (Additional file 2: Table S1).

**Table 1:** Characteristics of study patients at enrollment

Characteristics at enrollment	Included group (***N*** = 63)	Excluded group (***n*** = 40)	Total (***n*** = 103)
Age (years)	11 (8, 14)	12 (8, 13)	11 (8, 14)
Female	27 (43)	22 (55)	49 (48)
Weight (kg)	40 (30, 49)	39 (30, 50)	39 (30, 50)
Day of illness at enrolment	6 (5, 6)	5 (4, 5)	5 (4, 6)
Heart rate	104 (92, 119)	100 (92, 120)	100 (92, 120)
SBP	100 (90, 100)	100 (90, 105)	100 (90, 100)
DBP	80 (70, 80)	80 (64,80)	70 (65, 80)
Temperature (°C)	37 (37, 37.5)	37 (37, 37)	37 (37, 37)
Respiratory rate (breaths/min)	20 (20, 24)	20 (20, 24)	20 (20, 24)
Pulse pressure ≤ 10 mmHg	5 (8)^a^	1 (0)	6 (0.1)
Hematocrit (%)	48 (46,52)	50 (46, 52)	49 (46, 52)
Platelet at enrolment (× 1000 cells/μL)	27 (17, 35)	32 (19, 47)	29 (18, 41)
WBC (k/μL)	4.40 (3.14, 6.28)	4.61 (3.07, 5.75)	4.59 (3.11, 6.05)
AST (IU/L)	196 (114, 464)	142 (90, 251)	165 (104, 378)
ALT (IU/L)	100 (56, 189)	68 (41,116)	91 (44, 172)
Venous lactate (mmol/L)	3.0 (2.2, 4.4)	2.7 (2.2, 3.7)	2.9 (2.2, 4.1)

Statistics presented: median (IQR), *n* (%)

^a^There were 4 cases with pulse pressure ≤ 10 mmHg and 1 case with unmeasurable blood pressure

*SBP* systolic blood pressure, *DBP* diastolic blood pressure, *WBC* white blood cell, *AST* aspartate aminotransaminase, *ALT* alanine transaminase

### Dynamic changes of CRI during 48-h fluid resuscitation in ICU

**Fig. 1:**
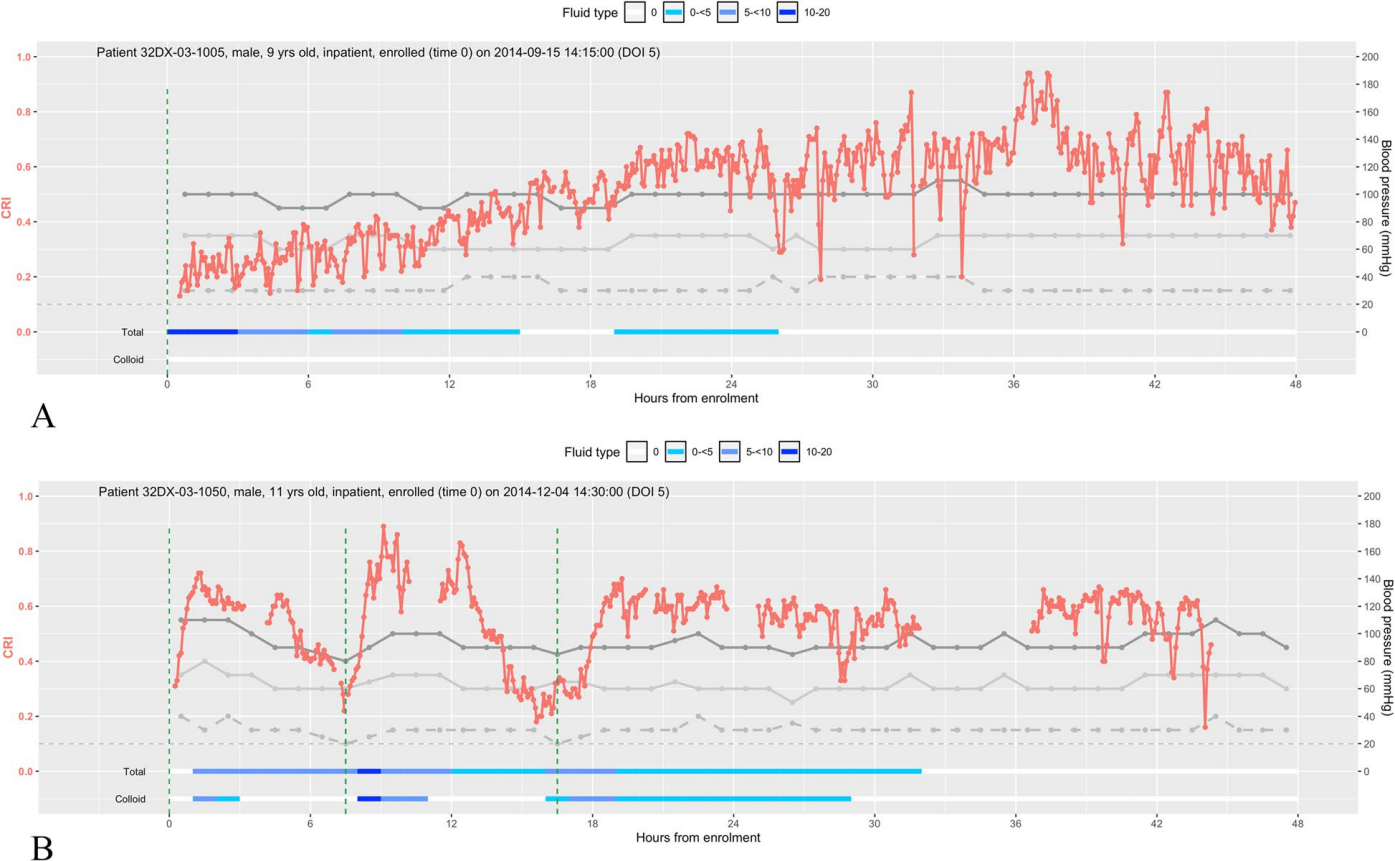
Dynamic changes in the compensatory reserve index (CRI), systolic blood pressure (SBP), diastolic blood pressure (DBP) and pulse pressure (PP) during the first 48 h of fluid management. **A** The trajectories of CRI, SBP, DBP, and PP in a 9-year-old boy with DSS. **B** The trajectories of CRI, SBP, DBP, and PP in an 11-year-old boy with DSS and two re-shock episodes. Red line is CRI, dark grey line is SBP, light grey line is DBP, intermittent grey line is PP, and the vertical green line indicates episodes of clinical shock/re-shock

Figure 1 shows the dynamic changes in CRI and BP in two illustrative cases with 48 h of monitoring, including changes in resuscitation fluid volumes. These two cases demonstrate the different trajectories of CRI over the first 48 h of fluid resuscitation and the association between CRI with systolic BP, diastolic BP and PP. Values of CRI fluctuate, but the overall trend reflects the changes in fluid resuscitation. The first case (Fig. 1A) is that of a 9-year old boy who received only crystalloid fluid (Ringers Lactate); the CRI gradually improved with fluid infusion, and there were no episodes of re-shock. The second case is that of an 11-year-old boy who presented with severe hypotensive shock and received colloid (hydroxyethyl starch 6% 200/0.5) for initial fluid resuscitation. The CRI increased sharply after an hour-long colloid infusion to reach the normal range, but fell again, heralding an episode of re-shock. Additional file 1: Fig. S2 is a pictorial description of all 63 patients included in the final analysis, showing their CRI trajectories, pulse pressure, and episodes of shock during the first 48 h from enrolment.

### Correlation between CRI and heart rate and systolic and diastolic blood pressure

**Fig. 2:**
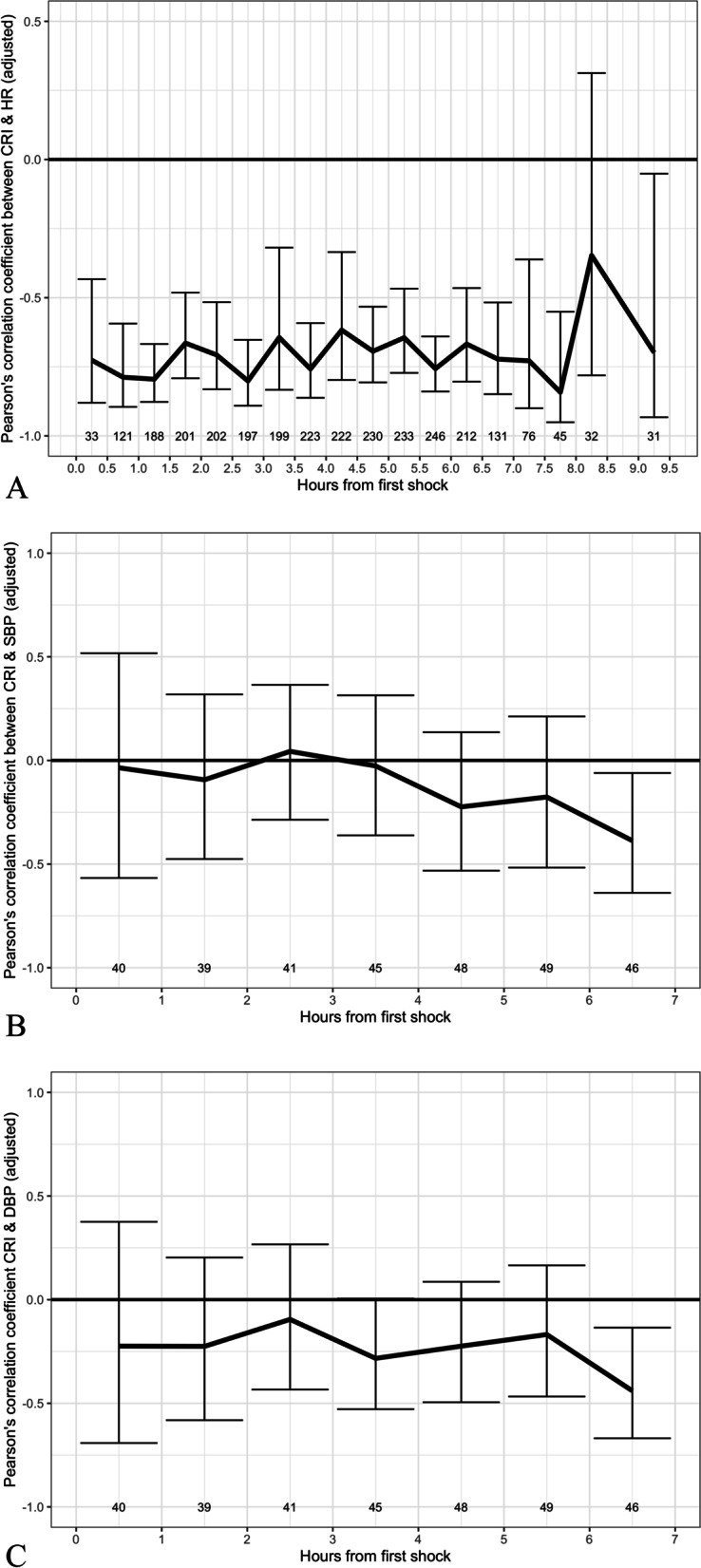
Pearson’s correlation coefficient between compensatory reserve index (CRI) and heart rate (HR), systolic blood pressure (SBP) or diastolic blood pressure (DBP). The three panels describe partial Pearson’s correlation coefficients between CRI and heart rate (HR) (**A**), systolic blood pressure (SBP) (**B**) and diastolic blood pressure (DBP) (**C**) for all 63 cases included in the analysis. Solid black lines represent the estimated partial Pearson’s correlation coefficients between CRI values and the corresponding hemodynamic parameter at intervals from onset of the first shock episode, after adjusting for age, gender, and body weight. To perform these calculations, data within the first 8 h since the first shock were grouped every 30 min (for HR) or 1 h (for SBP and DBP). Vertical lines are corresponding 95% confidence intervals of the estimated partial Pearson’s correlation coefficient; repeated data was accounted for using bootstrap sampling. Number below each vertical line represents number of hemodynamic parameter measurements in each group (HR in **A**, SBP in **B**, and DBP in **C**). Correlations based on small numbers of observations (< 30) are unreliable and therefore they were excluded in these figures (> 9.25 h for HR, > 6.5 h for SBP and DBP)

Figure 2A–C shows a negative correlation between CRI and heart rate, SBP, and DBP within 8 h of the first episode of shock. CRI has a significant negative association with heart rate [overall partial Pearson’s correlation coefficient = − 0.71 (95% CI − 0.80, − 0.60)], which is stable within the first 9 h since the initial shock (Figure 2A). CRI has a weak negative correlation with SBP and DBP [overall partial Pearson’s correlation coefficients are − 0.12 (95% CI − 0.31, 0.07) and − 0.20 (95% CI − 0.38, 0)]. In general, associations between CRI and heart rate/blood pressure is relatively constant within the first 9 h of the observation from the first shock.

### CRI and prediction of re-shock and narrow pulse pressure

A decrease in CRI had a strong positive relationship with episodes of re-shock within 6 h [adjusted OR of 2.31 (1.40–3.81, *P* = 0.001)] and 12 h [adjusted OR of 2.24 (1.34–3.73, *P* = 0,002)] of measurement (Table 2). A decrease in CRI also had a positive relationship with the occurrence of a narrow pulse pressure (a key criterion for defining DSS) within 6 h of measurement, with an adjusted OR of 1.33 (0.96–1.84, *P* = 0.083) but not within 12 h of measurement (Table 2). Of note, the majority of first re-shocks occurred within 12.0 h (IQR 11.5, 13.7 h) of enrolment and 5.4 h (IQR 2.9, 6.8 h) of first CRI measurement after cardiovascular stability (Additional file 2: Table S2). Overall, the CRI was able to predict re-shock within 12 h of measurement with an AUC of 0.84 (95% CI 0.75–0.93). A CRI cutoff of 0.4 provided the best sensitivity (0.66 [95% CI 0.47–0.85]) and specificity (0.86 [95%CI 0.80–0.92]) (Table 3). In patients with re-shock, the mean CRI decreased below 0.4 from 2 h prior to the event (Additional file 1: Fig. S3). This was in contrast to those patients who never developed an episode of re-shock; their mean CRI remained consistently above 0.4 (Additional file 1: Fig. S4).

**Table 2:** Prediction of first episode of re-shock (or narrow pulse pressure) using CRI measurement

	Within 12 h of CRI measurement		Within 6 h of CRI measurement	
	Estimate (95%CI)	***P***	Estimate (95%CI)	***P***
**First re-shock**				
OR adjusted	2.24 (1.34, 3.73)	0.002	2.31 (1.40, 3.81)	0.001
OR unadjusted	2.17 (1.42, 3.32)	< 0.001	2.25 (1.55, 3.28)	< 0.001
**Narrow pulse pressure**				
OR adjusted	1.24 (0.82, 1.86)	0.306	1.33 (0.96, 1.84)	0.083
OR unadjusted	0.98 (0.34, 2.77)	0.963	1.43 (1.01, 2.02)	0.045

For each outcome (first re-shock and narrow pulse pressure), we considered two risk periods: within 6 h from CRI measurement and within 12 h from CRI measurement

*OR* odds ratio of the first re-shock or narrow pulse pressure for each decrease of 0.1 in CRI. These ORs and corresponding 95% confidence intervals (95%CI) and *P* values were estimated from logistic regression models that used cluster bootstrap with 1000 resamples of the original dataset to take into account repeated CRI measurements from the same participant

*OR adjusted* corresponds to OR after adjusted for time of CRI measurement, age, gender, and body weight

*OR unadjusted* corresponds to OR from univariate model with CRI as the only covariate

**Table 3:** Performance of different CRI cutoffs in predicting first episode of re-shock

Measure	Within 12 h of CRI measurement	Within 6 h of CRI measurement
	Estimate (95%CI)	Estimate (95%CI)
AUC	0.84 (0.75, 0.93)	0.86 (0.77, 0.94)
CRI cutoff 0.2		
Sensitivity	0.19 (0.02, 0.36)	0.22 (0.02, 0.42)
Specificity	0.98 (0.97, 0.99)	0.98 (0.97, 1.00)
CRI cutoff 0.4		
Sensitivity	0.66 (0.47, 0.85)	0.68 (0.49, 0.88)
Specificity	0.86 (0.80, 0.92)	0.86 (0.80, 0.91)
CRI cutoff 0.6		
Sensitivity	0.93 (0.84, 1.00)	0.95 (0.90, 1.00)
Specificity	0.51 (0.42, 0.59)	0.50 (0.42, 0.58)

*AUC* area under the ROC curve

*95%CI* 95% confidence intervals were calculated using cluster bootstrap standard errors based on 1000 resamples of the original dataset

These results were based on CRI, without any adjustment and did not correct for optimism

## Discussion

We have shown that CRI derived from continuous, non-invasive algorithmic analysis of the pulse waveform in children and adults with severe dengue can predict the onset of shock. The CRI was able to predict an episode of re-shock within 12 h from measurement and predicted a narrowed pulse pressure (PP ≤ 20 mmHg)—a key diagnostic criterion of DSS—within 6 h (Table 2). We identified a CRI cutoff value of 0.4 as providing the best sensitivity and specificity, when distinguishing patients who developed re-shock from those that did not—a finding that held true up to 2 h prior to the event (Table 3, Additional file 1: Fig. S3 and Fig. S4). In our analyses, we show that the CRI had negative correlation with heart rate and systolic and diastolic blood pressure for the period of monitoring. As fluid leakage occurs in an individual, the CRI decrease is accompanied by a rise in heart rate, but also a rise in diastolic blood pressure due to compensatory vasoconstriction in the period prior to circulatory collapse. These results suggest that CRI could be a potentially helpful monitoring tool for the management of DSS in healthcare settings around the world, where obtaining contemporaneous vital signs, the assessment of intravascular volume of individual patients, and trending their responses to fluid resuscitation remain as huge challenges due to workload and limited healthcare resources.

The exaggerated inflammatory response in severe dengue results in a profound vascular leakage syndrome and shock [18]. The ability to provide judicious fluid therapy is therefore crucial and needs to be balanced with the risk of excessive fluid replacement, leading to complications of volume overload and respiratory distress [19]. Changes in conventional vital signs occur late in the setting of shock and lack specificity [8], and the use of cuff-based or invasive blood pressure monitoring techniques in severe dengue can result in bleeding as a result of profound thrombocytopenia. These modalities also require a level of healthcare expertise and infrastructure often unavailable in resource-limited settings—frequent, repeated vital signs measurements for patients in ward settings for example is often challenging. The CRI operates through photoplethysmography-based pulse waveform analysis—similar to pulse oximetry [8]. CRI has been clinically validated in severe trauma [20], and there is evidence that an individualized assessment of volume status is provided [21]. In our study, we adopted re-shock as our endpoint because it is a robust clinical endpoint that is widely used and clinically meaningful—the onset of shock prompts additional fluid replacement and interventions. Pulse pressure (PP) was used as a secondary endpoint, because a narrowed PP is a key diagnostic criterion for DSS, according to the WHO classification [7].

Traditional vital sign monitoring such as heart rate is an indicator of hemodynamic status, but can be affected by many factors, including temperature, stress, arrhythmias, and pain [7, 22], and the relative bradycardia observed in dengue patients could mask deterioration [23], so more robust ways of monitoring hemodynamic status are required. Other studies show that CRI is a more sensitive and specific indicator of decreased central blood volume status when compared to heart rate, blood pressure, SpO_2_, lactate, perfusion index, tissue oxygenation, etc. [8–10, 20, 24–29]. In dengue endemic settings such as Vietnam, it is essential that any technological intervention be robust, accepted by patients, cost-effective and adaptable to existing workflows—monitoring through PPG fulfills these criteria due to its non-invasive nature, relatively low costs, and staff familiarity with the use of pulse oximetry [30]. The use of CRI and other PPG-based monitoring devices could enable rapid risk-stratification and triage of patients most at risk, prior to overt clinical deterioration. In our study, patients were connected to a finger probe with data transferred wirelessly to a laptop. A standalone CRI monitor is FDA-cleared and commercially available; however, integration into bedside multi-parameter monitors would be more practical. The ability of the CRI to provide a continuous real-time assessment of cardiovascular status additionally could be promising for guiding individualized fluid management and allows for the development of novel algorithms which are dynamic and responsive to patient clinical state. We observed changes in CRI values in response to fluid therapy (Fig. 1), suggesting a benefit of CRI monitoring not only to detect shock but also to evaluate the response to fluid during resuscitation and guide further interventions. Work in coupling intrinsic connectivity [31] and automated alerts to the end-user could greatly improve caseload management in austere healthcare settings and in the context of epidemics [32]. This work currently forms an active area of research within our group [33].

The strengths of the study include its prospective design, the use of clinically relevant endpoints, and large patient sample size with the use of prolonged continuous CRI data to construct prediction models. Limitations include exclusion of enrolled patients because of inadequate or incomplete CRI data—this reflects the nature of PPG, which can be influenced by motion artifact and poor skin perfusion, which is relevant when monitoring patients in shock. A comparison with other measurements of fluid responsiveness, such as echocardiography, was not feasible at the time of this study but warrants investigation. We were also not able to directly compare the predictive performance of the CRI with that of other vital signs (such as heart rate and blood pressure) given the study design, in which the endpoint of shock used is itself defined by the pulse pressure. Ideally, a randomized head-to-head study allocating patients into different monitoring groups would allow for more robust analyses. Finally, as an observational study, we could not assess the role of CRI in terms of its actual clinical utility—we speculate that the automated and continuous nature of patient monitoring is scalable in healthcare settings and can provide useful information to guide clinicians in triage and treatment. The characterization of utility metrics in the healthcare system beyond its predictive value and establishing its role in patient management compared with standard practice is now needed.

## Conclusions

Non-invasive pulse waveform measurement through the CRI is a useful method for assessing intravascular volume status in patients with DSS. It is able to predict the onset of re-shock in severe dengue within 12 h of measurement, with a CRI cutoff of 0.4 providing the best sensitivity and specificity. CRI can assist in the early detection of re-shock in dengue patients admitted to ICU and could have a role in guiding individualized fluid resuscitation strategies in these patients.

## Supplementary Information

**Additional file 1: Fig. S1**. Flowcharts of included patients. **Fig. S2.** Trajectory of CRI (red line) and pulse pressure (black line) during 48 h in pediatric intensive care unit form enrolment of 63 study patients with dengue shock syndrome (DSS). **Fig. S3**. Mean compensatory reserve index (CRI) and heart rate (HR) at different time-point prior to re-shock. **Fig. S4**. Mean compensatory reserve index (CRI) and heart rate (HR) at different time-points from onset of the initial shock episode among the 47 patients who never developed re-shock.

**Additional file 2: Table S1**. Parenteral fluid management and outcomes of study patients included in the final analysis. **Table S2**. Duration from initial CRI measurement to first re-shock.

## Data Availability

The datasets generated and/or analyzed during the current study are available in the Oxford Research Archive for Data (ORA-data) repository, 10.5287/bodleian:EoxBbEQk4 [34].

## Abbreviations

AUC: Area under the curve
AICU: Adult intensive care unit
CRI: Compensatory reserve index
DBP: Diastolic blood pressure
DSS: Dengue shock syndrome
HTD: Hospital for Tropical Diseases
HR: Heart rate
OR: Odds ratio
PICU: Pediatric intensive care unit
PPG: Photoplethysmography
SBP: Systolic blood pressure
