# Supplementary material for: The compensatory reserve index predicts recurrent shock in patients with severe dengue
Source: BMC Med. 2022 Apr 7;20:109. doi: 10.1186/s12916-022-02311-6 (PMC8986451; doi:10.1186/s12916-022-02311-6)

**Additional file 1**

**Fig.S1. Flowcharts of included patients**

AICU: Adult Intensive Care Unit. PICU: Paediatric Intensive Care Unit.

**Enrolment N= 103**

AICU 19, PICU 84

**Data assessment N= 96**

AICU 19, PICU 77

**Final analysis N=63**

AICU 13, PICU 50

Exclude:

- 5 cases : no CRI data
- 1 case not confirmed dengue
- 1 case: no clinical data

Exclude 33 cases

- 17 did not have shock
- 10 developed first shock after enrolment
- 6 did not have CRI data within the first 48hours


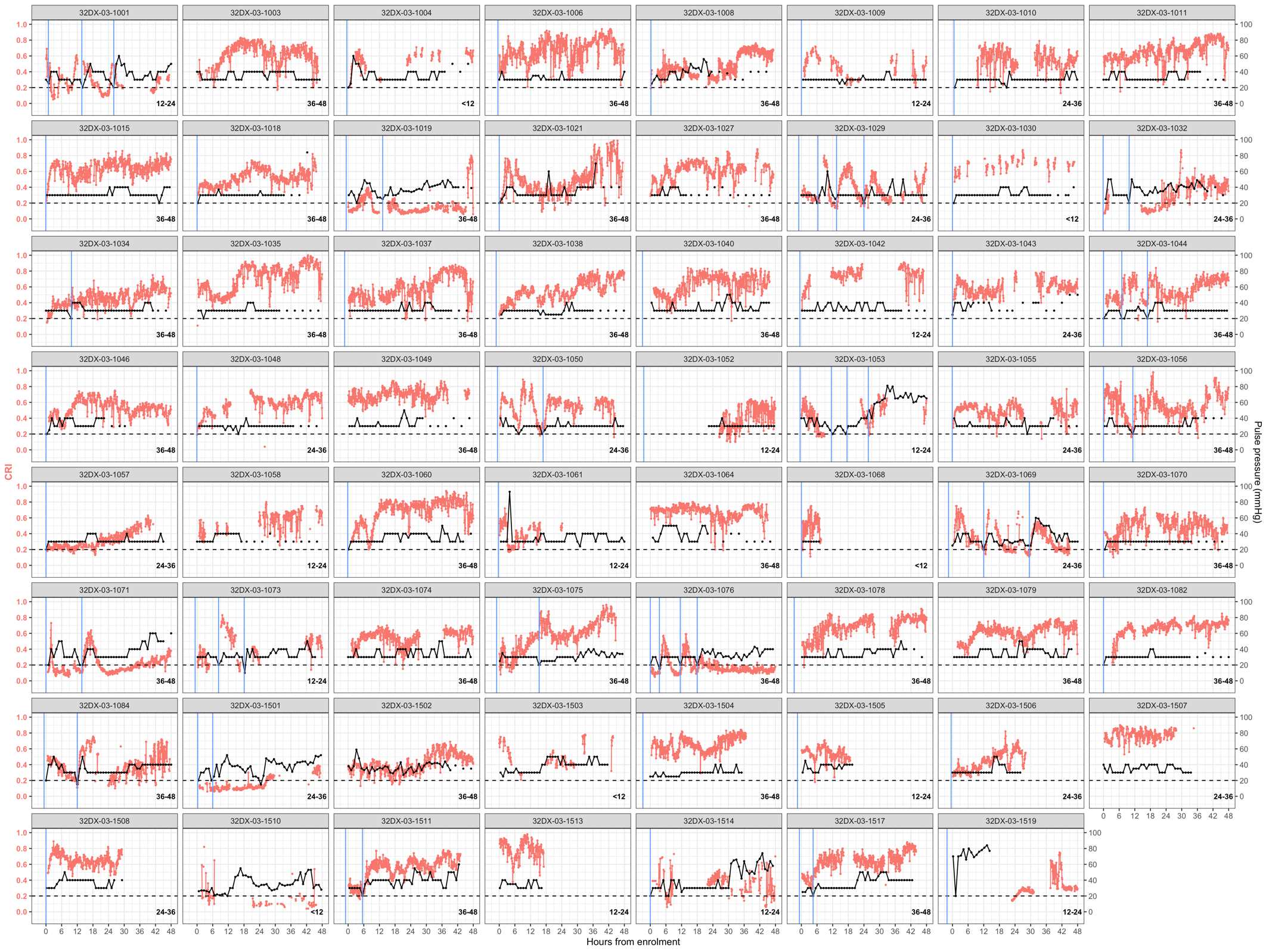


**Fig. S2. Trajectory of CRI (red line) and pulse pressure (black line) during 48 hours in paediatric intensive care unit form enrolment of 63 study patients with dengue shock syndrome (DSS)**. Intermittent black line is the indicator line for pulse pressure of 20 mmHg. Vertical blue line is indicator of shock based on clinical decision. A patient with DSS may have one or more episodes of shock during 48hours of fluid management. A clinical decision of shock is based on pulse pressure <=20 mmHg and signs of low peripheral perfusion with changes in fluid type (using colloids) and rates (≥ 10 ml/kg/h).

**Fig. S3. Mean compensatory reserve index (CRI) and heart rate (HR) at different time-point prior to re-shock**

Dots and lines represent the means and corresponding 95% confidence intervals for CRI (in red) and heart rate (in blue) at different time-points before the first re-shock occurrence for the 13 patients with re-shock, and the overall means and 95% confidence interval within the first 48 hours for the 47 patients without re-shock. Data were grouped within sequential 1-hour windows leading up to the first re-shock before calculation and 95% confidence intervals were estimated using the bootstrap standard errors based on 1000 resamples of the original dataset (the resampling accounts for repeated measurements from the same participant). Only CRI values measured after haemodynamic stability were included in this analysis. Numbers at bottom present number of CRI or HR measurements (upper) and number of patients (lower) in each group.


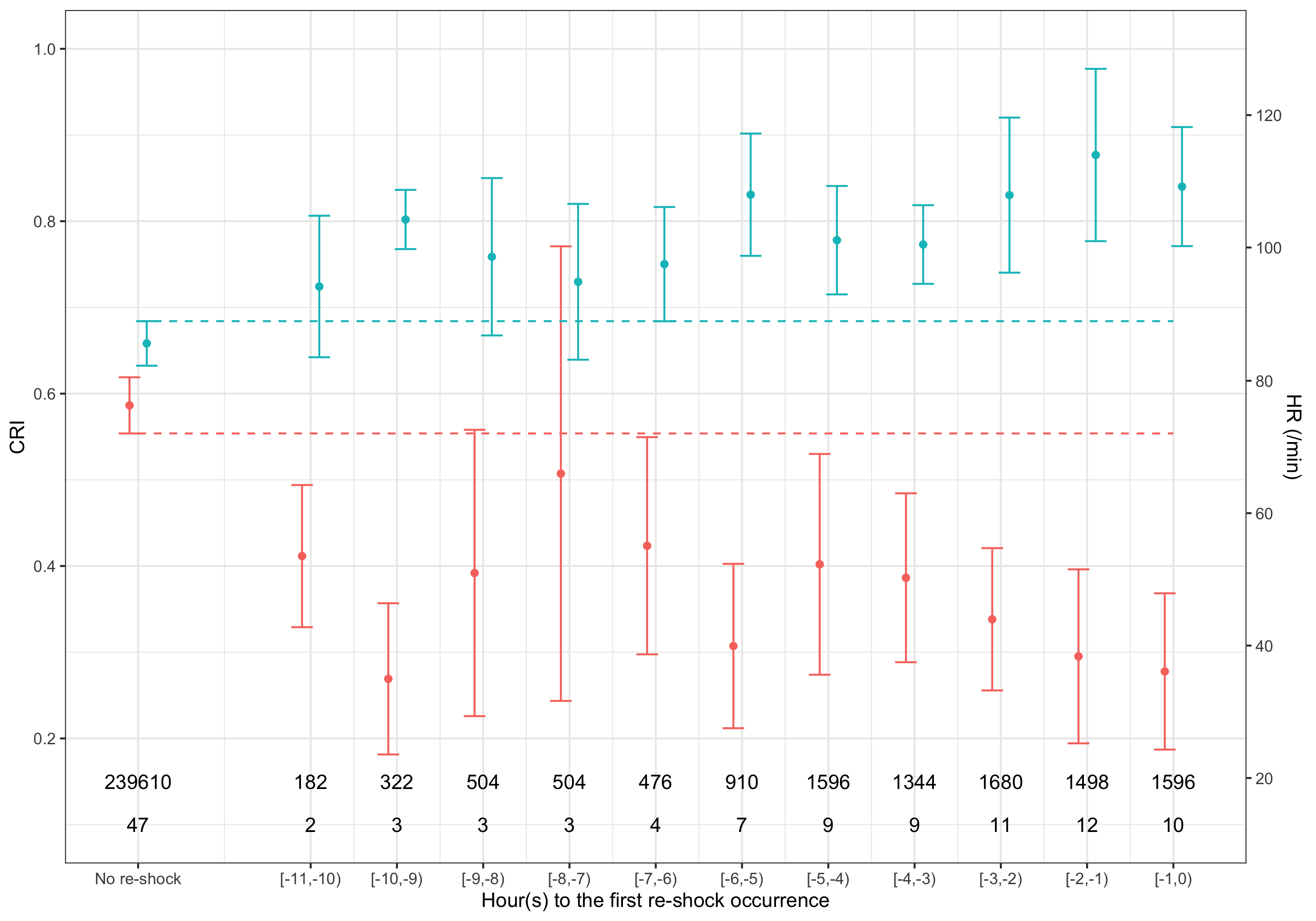


**Fig S4. Mean compensatory reserve index (CRI) and heart rate (HR) at different time-points from onset of the initial shock episode among the 47 patients who never developed re-shock.**

Dots and lines represent the means and corresponding 95% confidence intervals for CRI (in red) and heart rate (in blue) at different time-points from onset of the initial shock at enrolment. Data were grouped within sequential 1-hour windows after the first shock onset before calculation and 95% confidence intervals were estimated using the bootstrap standard errors based on 1000 resamples of the original dataset (the resampling accounts for repeated measurements from the same participant). Numbers at bottom present number of CRI or HR measurements (upper) and number of patients (lower) in each group.


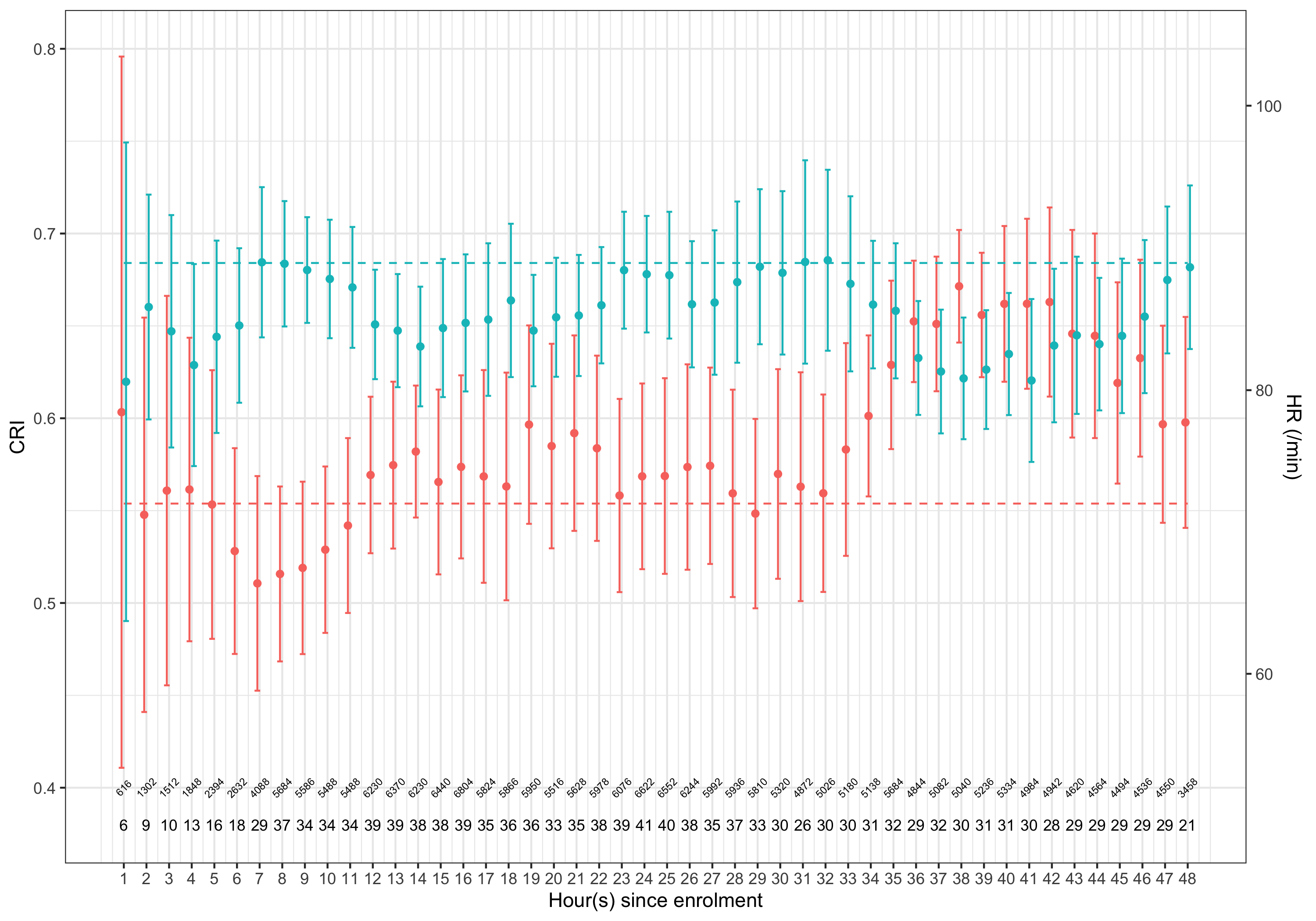

Supplement: Supplementary file 1 — Additional file 1: Fig. S1. Flowcharts of included patients. Fig. S2. Trajectory of CRI (red line) and pulse pressure (black line) during 48 h in pediatric intensive care unit form enrolment of 63 study patients with dengue shock syndrome (DSS). Fig. S3. Mean compensatory reserve index (CRI) and heart rate (HR) at different time-point prior to re-shock. Fig. S4. Mean compensatory reserve index (CRI) and heart rate (HR) at different time-points from onset of the initial shock episode among the 47 patients who never developed re-shock. [file 12916_2022_2311_MOESM1_ESM.docx]
