# Supplementary material for: The compensatory reserve index predicts recurrent shock in patients with severe dengue
Source: BMC Med. 2022 Apr 7;20:109. doi: 10.1186/s12916-022-02311-6 (PMC8986451; doi:10.1186/s12916-022-02311-6)
Supplement: Supplementary file 2 — Additional file 2: Table S1. Parenteral fluid management and outcomes of study patients included in the final analysis. Table S2. Duration from initial CRI measurement to first re-shock. [file 12916_2022_2311_MOESM2_ESM.docx]

**Additional file 2**

**Table S1. Parenteral fluid management and outcomes of study patients included in the final analysis**

| **Management and outcomes** |  | Median (IQR) or n (%)  N= 63 |
| --- | --- | --- |
| Duration of fluid management (hours) |  | 26 (22, 31) |
| Total volume of fluid resuscitation (ml/kg) |  | 112 (78,143) |
| Hours from onset of shock to initial haemodynamic stability |  | 7.0 (6.5, 10.7) |
| Re-shock |  | 15 (24) |
| Number of episodes  1 time  2 times  3 times |  | *10 (48)*  *4 (27)*  *1 (7)* |
| Hours from enrolment to first re-shock |  | 12 (10, 14) |
| Respiratory distress* |  | 8 (13) |
| Severe bleeding** |  | 4 (6) |
| Deaths |  | 0 (0) |

Statistics presented: median (IQR), n (%)

*Respiratory distress was defined clinically if having signs of using accessory muscles and/or requiring respiratory support. ** Severe bleeding was defined as bleeding required blood transfusion or procedures to stop bleeding.

**Table S2. Duration from initial CRI measurement to first re-shock**

| **Variable** | **Median Number of Hours (Interquartile range) [min, max]** | **Number of Episodes or Observations**  **(n=13)** |
| --- | --- | --- |
| Hours from enrolment to the first re-shock | 12.0 (11.5, 13.7) [6.3, 16.8] | 13 |
| Hours from the first CRI measurement to the first re-shock * | 5.4 (2.9, 6.8) [1.3, 11.3] | 13 |
| Hours from any CRI measurement to the first re-shock ** | 3.4 (1.8, 5.2) [0.0008, 11.3] | 10668 |

3/63 patients were excluded from the analysis of first re-shock prediction as there were no CRI measurements within the at-risk period (time period from hemodynamic stability to first re-shock or end of the 48-hour period).

* First CRI measurement is taken from the at-risk period after haemodynamic stability (excluding measurements when patients were still unstable from the first shock). This duration corresponds to the maximum time from any CRI measurement to first re-shock per patient.

** This time period includes every CRI measurement within the at-risk period to first re-shock; this accounts for all CRI measurements per patient.
